# Early-Life High-Fat Diet Impairs Autophagy and Reduces GABA_A_ Receptor Expression in Hypothalamic Neurons, Promoting Depressive-like Behaviors in Offspring Mice

**DOI:** 10.3390/nu18142312

**Published:** 2026-07-14

**Authors:** Muzi Qian, Ogula Doubra, Yadi Su, Shuyu Bai, Han Zhou, Hong Li, Dongying Yan, Liang Gao

**Affiliations:** 1 School of Public Health, Jinzhou Medical University, Jinzhou 121001, China; 17719349211@163.com (M.Q.); 19275579977@163.com (O.D.); 17719349211@126.com (Y.S.); z13956886916@126.com (S.B.); xiaobaiwangwang@yeah.net (H.Z.); lihongllll@126.com (H.L.); 2 Collaborative Innovation Center for Health Promotion of Children and Adolescents, Jinzhou Medical University, Jinzhou 121000, China

**Keywords:** high-fat diet, depressive-like behavior, autophagy–GABARAP pathway, hypothalamic neurons, GABA_A_ receptor

## Abstract

Backgrounds: Early-life nutritional excess increases the risk of depressive-like phenotypes in offspring, yet the underlying mechanisms remain unclear. This study investigated whether perinatal and adolescent high-fat diet (HFD) exposure is associated with depressive-like behaviors through disruption of the autophagy–GABARAP pathway in hypothalamic neurons. Methods: A mouse model of HFD exposure during both periods was established. Depressive-like behaviors, hypothalamic autophagic flux, and GABARAP–GABA_A_R co-localization were assessed. Primary hypothalamic neuronal cultures were treated with rapamycin. RNA sequencing and co-immunoprecipitation were performed to identify transcriptional changes and protein–protein interactions. Results: HFD exposure induced metabolic disturbances and depressive-like behaviors, suppressed autophagic flux (p62 and LC3-II accumulation), increased GABARAP–GABA_A_R co-localization, and reduced GABA_A_R protein abundance with elevated hypothalamic neuronal activity. Rapamycin reversed these alterations in primary neurons. RNA sequencing identified 87 differentially expressed genes (e.g., upregulated Plin4, Txnip; downregulated Npas4, Avp), while GABA receptor subunits and core autophagy genes remained unchanged at the transcriptional level. Enrichment analyses linked differentially expressed genes to mitophagy, oxidative phosphorylation, and the ubiquitin–proteasome system, with downregulated neuronal pathways. Co-immunoprecipitation confirmed direct GABARAP–GABA_A_R interaction. Conclusions: Impaired autophagic flux correlates with reduced GABA_A_R protein levels and increased hypothalamic neuronal activity, suggesting a link between autophagy–GABARAP dysregulation and depressive-like behaviors. The autophagy–GABARAP axis, together with transcriptomic reprogramming, may represent a key link between early-life nutritional excess and offspring depressive-like phenotypes, warranting further investigation as an intervention target.

## 1. Introduction

Depression is a critical mental health threat for children and adolescents, ranking among the most prevalent and debilitating psychiatric disorders in pediatric and youthful populations [1,2]. According to the Global Burden of Disease research findings, depression stands as the primary cause of illness-induced disability in individuals aged 10 to 19 years [3,4], and constitutes the third leading factor contributing to disability-adjusted life years (DALYs) in this age bracket. In severe clinical cases, depression can further trigger suicidal behaviors, which currently ranks fourth among the leading causes of death for people aged 15–29 years [5]. The latest epidemiological surveys have demonstrated that the global prevalence of mild to severe depression among children and adolescents is as high as 21.3% [6].

Based on the Developmental Origins of Health and Disease (DOHaD) theoretical framework, accumulating clinical and preclinical evidence confirms that adverse gestational maternal conditions markedly elevate the risk of neurodevelopmental abnormalities and psychiatric disorders in offspring [7,8]. In such adverse prenatal factors, maternal obesity induced by high-fat diet (HFD) intake has attracted extensive research attention. Non-human primate studies have verified that maternal HFD consumption during pregnancy can trigger anxiety and depression-like behavioral phenotypes in progeny [9,10,11]. Despite growing evidence supporting the biological correlation between maternal obesity and offspring depression, the exact molecular mechanisms by which maternal obesity modulates juvenile emotional behaviors remain poorly elucidated.

Hypothalamic agouti-related protein (AgRP) neurons are well-established core regulators of appetite and feeding behaviors, exerting modulatory effects via neural projections to multiple brain regions including the hypothalamic paraventricular nucleus (PVH), lateral hypothalamus (LH), and parabrachial nucleus (PBN) [12,13]. Emerging studies have further expanded the physiological functions of AgRP neurons, indicating that their neuronal activity not only dominates systemic energy homeostasis, but also participates in the regulation of circadian rhythms, pain sensation, and stress responses [14,15]. Notably, chronic stress exposure suppresses AgRP neuronal activity, and such inhibitory alterations are closely correlated with the emergence of depression-like behaviors [16]. This suggests that AgRP neural circuits may act as a critical integration hub linking metabolic signaling pathways and emotional regulatory functions [17]. Therefore, exploring the functional role of AgRP neurons in metabolism-associated mood disorders is essential for uncovering the pathogenic mechanisms of juvenile depression. However, it is important to note that the present study utilized whole hypothalamic tissue and mixed primary hypothalamic cultures, and therefore does not directly test AgRP neuron-specific effects.

As a typical subpopulation of GABAergic neurons, AgRP neurons mediate rapid synaptic transmission mainly by releasing gamma-aminobutyric acid (GABA), the predominant inhibitory neurotransmitter in the central nervous system [18,19]. Dysfunction of GABAergic signaling is a classic pathological feature of depression. Consistent clinical findings have verified reduced GABA levels and downregulated GABA_A_ receptor expression in the cerebral cortex of depressed patients. GABA_A_ receptors (GABA_A_Rs) are fast-acting inhibitory ion channels, and their functional expression level directly determines the efficiency of inhibitory synaptic transmission [20,21]. In the context of energy metabolism, GABA_A_R signaling is also indispensable: pharmacological activation of GABA_A_Rs mimics the hyperphagic phenotype induced by AgRP neuron activation, while pharmacological blockade reverses this abnormal feeding behavior [22]. These findings indicate that the proper functional expression of GABA_A_Rs in hypothalamic neurons participates in the dual regulation of metabolic homeostasis and emotional processing. The functional availability of GABA_A_Rs is modulated by multiple auxiliary proteins, among which the GABA_A_ receptor-associated protein (GABARAP) has become a key research focus [23]. Initially identified as a binding partner of the GABAAR γ2 subunit, GABARAP has been reported to regulate the intracellular localization and surface expression of GABA_A_Rs in overexpression or heterologous systems [24]. Subsequent studies have further classified GABARAP as a member of the Atg8 family of core autophagy proteins, which plays a vital role in autophagosome formation and maturation [25]. Autophagy is a fundamental cellular homeostatic mechanism that eliminates damaged proteins and dysfunctional organelles to maintain cellular physiological stability [26]. Recent studies have revealed that GABARAP can bind to the upstream autophagy kinase ULK1 and the downstream adaptor protein PLEKHM1, serving as a scaffold molecule to regulate multiple stages of autophagic flux [27]. These research advances raise a key scientific question: under pathological stress conditions, whether altered autophagic activity affects GABA_A_R protein levels or binding properties through changes in GABARAP function, thereby contributing to emotional dysfunction.

Latest transcriptomic studies have begun to clarify the molecular alterations of hypothalamic neural circuits induced by early-life nutritional stress. Single-cell RNA sequencing (scRNA-seq) analyses have confirmed that maternal or early-life HFD exposure induces widespread transcriptional changes in hypothalamic AgRP neurons [28,29]. Importantly, differential genes induced by HFD intervention are significantly enriched in autophagy–lysosomal pathway-related terms, accompanied by coordinated downregulation of GABAergic synaptic transmission-related gene sets [30]. Nevertheless, the complete transcriptional regulatory network connecting early-life HFD stimulation, hypothalamic neuronal dysfunction and depression-like behaviors remains unclear. Previous transcriptomic analyses lacked precise cell-type specificity, and the interactive relationships between transcriptional remodeling, impaired autophagic flux and GABAergic signaling in the hypothalamus under early nutritional stress have not been fully clarified [31,32,33,34,35,36].

To fill the above research gaps, this study systematically characterizes the transcriptomic profiles of hypothalamic tissue following early-life HFD exposure, and further explores how transcriptional remodeling relates to autophagic and GABAergic signaling in the context of depressive-like behaviors in offspring. Specifically, we focus on examining the correlation between the autophagy–GABARAP axis and GABA_A_R protein levels in hypothalamic tissue, and characterize the association between early-life HFD intervention and alterations in this signaling pathway in relation to depressive phenotypes. This study aims to provide novel mechanistic insights and potential therapeutic targets for the prevention and clinical intervention of depression caused by early-life metabolic disorders.

It should be emphasized that the experimental materials used in this study were hypothalamic tissue and mixed primary hypothalamic neuronal cultures, rather than purified AgRP neurons. Therefore, the observed alterations in the hypothalamic autophagy–GABARAP pathway and their association with depressive-like behaviors reflect global changes at the hypothalamic tissue level rather than effects specific to any particular neuronal subtype. Future studies employing cell-type-specific approaches (e.g., AgRP neuron-specific genetic manipulation) are warranted to further validate the functional roles of this pathway in defined neuronal populations.

## 2. Materials and Methods

### 2.1. Animals

A total of 18 pregnant C57BL/6J mice (body weight: 18–22 g) were purchased from Vital River Laboratory Animal Technology Co., Ltd. (Beijing, China). All experimental animals were raised in a specific pathogen-free (SPF) animal facility with a standardized 12 h light/dark cycle, constant ambient temperature (22 ± 2 °C) and relative humidity (55% ± 5%). All mice had free access to standard food and sterile drinking water throughout the feeding period. The control diet (Research Diets D12450B) has an energy density of 3.85 kcal/g, consisting of 70% carbohydrates, 20% protein and 10% fat. The high-fat diet (Research Diets D12451) provides 4.73 kcal/g energy, with a nutritional composition of 35% carbohydrates, 20% protein and 45% fat [34,35].

For the primary neuronal culture experiments, three additional pregnant dams per maternal dietary group (*n* = 3 per group, total 6 dams) were used exclusively for pup collection, and these dams were not included in the behavioral or tissue-level analyses to avoid confounding. Thus, the total number of pregnant dams used in this study was 18 (12 for in vivo experiments + 6 for primary neuronal cultures).

Body weight, food intake and water consumption of offspring mice were recorded weekly from weaning (postnatal day 21, P21) to adolescence (P45). For these longitudinal measurements as well as for blood pressure and heart rate recorded across multiple time points, data were analyzed using repeated-measures two-way ANOVA with maternal diet and adolescent diet as between-subject factors and time as the within-subject factor. When the sphericity assumption was violated, Greenhouse–Geisser correction was applied. Post hoc comparisons at individual time points were performed using Bonferroni-adjusted *t*-tests only when the overall interaction or main effect reached statistical significance. After the completion of all behavioral tests at P45, mice were anesthetized and euthanized. Hypothalamic tissues were rapidly isolated on ice, snap-frozen in liquid nitrogen, and stored in a −80 °C ultra-low-temperature refrigerator for subsequent molecular biological detection.

All experimental operations strictly followed the principles of random grouping, blind testing and animal welfare ethics. The entire animal experimental protocol was reviewed and approved by the Animal Ethics Committee of Jinzhou Medical University (Approval No. 20250070).

### 2.2. Experimental Design

Twelve pregnant dams were randomly assigned to two maternal diet groups (*n* = 6/group) and fed control or high-fat diet during the perinatal period (GD0 to P21). Each dam produced 6–12 pups, yielding approximately 3–6 male pups per litter based on a ~1:1 sex ratio; only males were used. To balance litter effects, a maximum of 5 male pups were randomly selected per litter (or all if <5), generating approximately 71 male offspring across 12 litters. After weaning (P21), male offspring were divided by maternal diet, and within each maternal group further randomized to adolescent control or HFD (to P45), forming four groups: (1) G0−/−: perinatal control diet + adolescent control diet (control throughout both periods, serving as the normal control group); (2) G0−/+: perinatal control diet + adolescent HFD (HFD exposure only during adolescence); (3) G1+/−: perinatal HFD + adolescent control diet (HFD exposure only during the perinatal period, with recovery to control diet in adolescence); (4) G1+/+: perinatal HFD + adolescent HFD (HFD exposure throughout both periods, continuous HFD group). From each group, 6 animals were randomly selected using a random number table (*n* = 6/group, total 24) for formal in vivo experiments, with no more than 2 pups from the same litter per group. For behavioral tests, 5 animals/group were randomly selected (*n* = 5/group, total 20; the remaining 1/group reserved for histology); for Western blot and immunofluorescence, 4 animals/group (*n* = 4/group, total 16); for qPCR, 3 animals/group (*n* = 3/group, total 12). The remaining ~26–46 offspring were used for primary neuronal culture validation and supplementary experiments. All 24 animals survived to P45 without mortality or exclusion.

**Figure 1:**
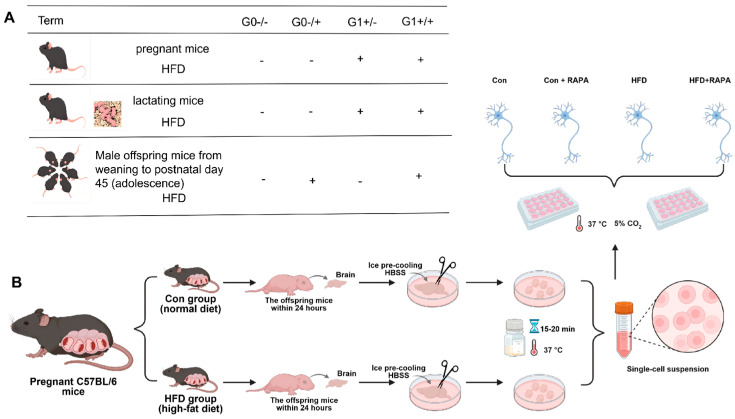
Schematic diagrams of animal grouping and primary cell isolation grouping. (**A**) In vivo animal experimental grouping design. Pregnant C57BL/6J dams were fed control or HFD during gestation. After birth, offspring from each maternal group received control or HFD during lactation (P0–P21), and were then rerandomized to control or HFD during adolescence (P21–P45). This generated four final groups: G0−/− (control throughout gestation, lactation, and adolescence); G0−/+ (control in gestation/lactation, HFD in adolescence); G1+/− (HFD in gestation/lactation, control in adolescence); G1+/+ (HFD throughout all periods). In the group labels, the first symbol refers to the perinatal period (gestation + lactation), and the second to adolescence; “+” denotes HFD exposure and “−” denotes control diet during the indicated period. (**B**) In vitro primary neuronal isolation and treatment grouping design.

For primary neuronal experiments, hypothalamic neurons were isolated from male neonatal offspring (within 24 h of birth) derived from dams fed control diet or HFD. Three independent litters were included per maternal dietary group, and two male pups were randomly selected from each litter, assigned to different treatment subgroups (vehicle control or 100 nM rapamycin) to avoid litter-dependent bias. After culture establishment, neurons were divided into four treatment groups: Con (control diet offspring neurons + DMSO), Con + RAPA (control diet offspring neurons + rapamycin), HFD (HFD offspring neurons + DMSO), and HFD + RAPA (HFD offspring neurons + rapamycin), with each group containing 3 independent litters (one pup/litter). A 2 × 2 factorial design (maternal diet × rapamycin) was adopted, with litter as a blocking factor. Adolescent HFD was not applied in vitro because (1) its systemic metabolic effects rely on intact in vivo circuits, (2) neonatal neurons have not experienced adolescent stimulation, and (3) rapamycin serves as an autophagy activator rather than a dietary mimetic. Grouping schematic is shown in Figure 1B. Hypothalami were dissected from P0 pups under a stereomicroscope using anatomical landmarks: rostral to the optic chiasm, caudal to the mammillary bodies, lateral to the hypothalamic sulci, and ~1 mm in depth. Tissues were digested with 0.25% trypsin-EDTA (25200-056, Gibco, Grand Island, NY, USA) at 37 °C for 15 min, terminated with HBSS containing DNase I and FBS, then triturated with fire-polished pipettes and filtered through a 70 μm strainer. Neurons from different pups were processed separately to ensure biological independence. Cells were seeded at 2 × 10^5^ cells/cm^2^ on 50 μg/mL poly-D-lysine-coated dishes in Neurobasal medium with 2% B27, 1% GlutaMAX, and 1% penicillin–streptomycin. Complete medium replacement was performed at 4 h, followed by half-volume changes daily. To inhibit glial proliferation, 5–10 μM cytarabine was added on days 5–7 for 24 h. After cytarabine removal, neurons were treated with 100 nM rapamycin (R8781, Sigma-Aldrich, St. Louis, MO, USA) or DMSO vehicle for 24 h. Rapamycin, an mTOR inhibitor, induces autophagy as validated previously. Cells were then collected for western blotting, immunofluorescence, and co-immunoprecipitation to assess autophagic flux and GABARAP–GABA_A_R interactions.

These cultures represent mixed hypothalamic neuronal populations derived from whole hypothalamic dissections. No enrichment, isolation, or immunocytochemical identification of specific neuronal subtypes (including AgRP-positive neurons) was performed. Consequently, all findings derived from these cultures reflect responses of the heterogeneous hypothalamic neuronal population as a whole, rather than AgRP-neuron-specific effects.

To minimize carryover effects and ensure data reliability, all offspring were subjected to a battery of behavioral tests starting at 8–10 weeks of age, with tasks arranged in ascending order of stress intensity. The test sequence was as follows: open field test (OFT) to assess locomotor and exploratory activity; three-chamber social interaction test (SIT) to evaluate social preference and social novelty; marble-burying test (MBT) to assess repetitive/anxiety-like behavior; tail-suspension test (TST) to evaluate despair-like behavior; and forced swim test (FST) as the terminal stress paradigm. A minimum 24 h interval was maintained between consecutive tests, and the TST and FST were separated by at least 48 h to prevent habituation and cumulative stress effects. All tests were conducted during the light phase between 9:00 and 15:00, and behavior was recorded and analyzed using an automated tracking system. We acknowledge that reduced locomotor activity in HFD-exposed offspring may partly reflect increased body weight or metabolic alterations rather than a pure depression-like phenotype; this possibility is considered in the Section 4.

### 2.3. Blood Pressure and Heart Rate Measurement

Systolic blood pressure and heart rate of offspring mice were measured weekly from P21 to P45 using a non-invasive tail-cuff blood pressure detection system (BP-2000, Visitech Systems, Sunderland, MA, USA). All mice underwent 5 consecutive days of adaptive training before formal detection. During daily adaptation, mice were placed in a 37 °C constant-temperature metal fixator for 5 min to stabilize body temperature and adapt to the experimental environment without cuff inflation. For formal data collection, mice were fixed in the same device and rested for 5 min before cuff inflation and parameter recording. All measurements were performed at the same time period every day to eliminate the interference of circadian rhythm differences.

### 2.4. Open Field Test

The open field test was carried out in a quiet and dim experimental environment with a square open field arena (50 cm × 50 cm × 40 cm). Each mouse was placed in the center of the arena, and spontaneous locomotor activities were continuously recorded for 5 min via an automatic video tracking system (Shanghai Xinruan Information Technology Co., Ltd., Shanghai, China). The main behavioral parameters analyzed included total movement distance and average movement speed. To eliminate residual odor interference between different mouse tests, the arena was thoroughly wiped and disinfected with 75% ethanol after each trial.

### 2.5. Three-Chamber Social Interaction Test

The three-chamber social interaction device consists of a rectangular box (60 cm × 40 cm × 40 cm) divided into three equal independent compartments by detachable baffles (Shanghai Xinruan Information Technology Co., Ltd., Shanghai, China). The test was divided into two sequential stages. In the social preference stage, a wire mesh cage containing an unfamiliar conspecific mouse (S1) was placed in the right compartment, and an empty wire mesh cage (E) was placed in the left compartment. After removing the partition baffles, the test mouse was allowed to explore freely for 10 min. In the social novelty preference stage, a second unfamiliar mouse (S2) was placed in the previously empty wire cage, and the mouse’s exploratory behavior was recorded for another 10 min. The residence time of mice in each compartment was automatically counted by the tracking system. The social preference index (SPI) was calculated as (TS1 − TE)/(TS1 + TE), and the social novelty preference index (SNPI) was calculated as (TS2 − TS1)/(TS2 + TS1).

### 2.6. Forced Swim Test

The forced swim test was performed in a transparent glass cylinder (height: 25 cm, inner diameter: 10 cm) filled with 15 cm deep constant-temperature water (25 ± 1 °C) in a quiet dim room. Each mouse was gently placed into the water and allowed to swim freely for 6 min. Immobility behavior was defined as the mouse maintaining an upright floating posture, with only minimal limb movements to keep the head above water without active swimming struggles. The immobility duration within the test period was statistically analyzed by blinded observers based on recorded videos.

### 2.7. Tail Suspension Test

Adhesive tape was attached to the tail tip (approximately 1 cm from the end) of each mouse, and the mouse was suspended upside down at a height of 50 cm from the ground. The total test duration was 6 min. Two core indicators were recorded: the immobility latency (the time from the start of the test to the first continuous immobility state lasting ≥2 s) and the total immobility time in the last 4 min (3–6 min) of the test. Passive suspension without active struggling was defined as effective immobility behavior.

### 2.8. Marble Burying Test

The marble-burying test was conducted in a standard mouse breeding cage (42 cm × 28 cm × 17 cm) paved with 5 cm thick clean bedding. Twenty dark glass marbles (diameter: 1.5 cm) were evenly arranged on the bedding surface in a 4 × 6 grid pattern. Each mouse was placed in the corner of the cage and allowed to explore freely for 30 min. A trained observer blinded to experimental grouping recorded the number of buried marbles (marbles covered by more than two-thirds of bedding) and the duration of behavioral immobility (only respiratory movement without active physical activity).

### 2.9. Quantitative Magnetic Resonance (QMR) Analysis

Mouse body composition was detected using an EchoMRI-500 quantitative magnetic resonance system (EchoMRI, Houston, TX, USA). The instrument was calibrated with 38.4 g canola oil standard before each detection. Mice were placed in a cylindrical plastic holder and fixed with soft plastic inserts to reduce body movement during scanning. The supporting EMR-184 analysis software automatically measured fat mass, lean mass, free water mass and total water mass of each mouse.

For phenotypic statistical analysis, the relative body fat percentage was calculated as (fat mass/total body weight) × 100%. Lean mass data were recorded but not included in final statistical analysis, as no significant inter-group differences were observed and it was irrelevant to the core metabolic and behavioral indicators of this study. Free water and total water mass data were collected but not analyzed in this study, for the following reasons: (1) body hydration status was not the core research endpoint; (2) previous studies have confirmed that dietary intervention does not cause significant changes in mouse hydration status; (3) these indicators cannot provide effective Supplementary Information (Table S1) for core research conclusions and will interfere with result interpretation. Nevertheless, these parameters can be used to evaluate systemic metabolic changes in subsequent expanded studies.

### 2.10. Blood Collection and Measurement of Glucose and Lipid Parameters

All mice were fasted overnight for 14–15 h with free access to drinking water. Fasting blood glucose was detected via the buccal vein using a OneTouch Verio Flex™ blood glucose meter (LifeScan, Malvern, PA, USA) on the next morning. After euthanasia, terminal blood samples were collected by cardiac puncture into EDTA-containing vacuum blood collection tubes (5.4 mg/tube, Becton, Dickinson and Company, Franklin Lakes, NJ, USA). Blood samples were placed on ice for 3 h and centrifuged at 5000× *g* for 15 min at 4 °C to separate plasma. The isolated plasma was subpacked and stored in a −86 °C ultra-low-temperature refrigerator for subsequent detection.

Plasma triglyceride (TG), total cholesterol (TC), low-density lipoprotein cholesterol (LDL-C) and high-density lipoprotein cholesterol (HDL-C) levels were detected using a Hitachi 7180 automatic biochemical analyzer with matching detection kits (Biosino Bio-Technology and Science Incorporation, Beijing, China). All detection operations strictly followed the manufacturer’s standard protocols. Quality control serum samples were set in each batch of tests to ensure intra-batch and inter-batch detection accuracy.

### 2.11. Western Blotting

Approximately 50 mg of hypothalamic tissue was homogenized on ice in RIPA lysis buffer supplemented with protease inhibitor cocktail. Tissue homogenate was centrifuged to collect supernatant, and protein concentration was quantified via BCA assay. Equal amounts of protein samples were denatured by high-temperature boiling with loading buffer, then separated by SDS-PAGE electrophoresis (80 V for stacking gel, 120 V for separating gel). The separated protein bands were transferred to PVDF membranes via wet electrotransfer (constant current 300 mA, 4 °C).

Membranes were blocked with 5% skimmed milk at room temperature, then incubated with specific primary antibodies overnight at 4 °C. After thorough washing, membranes were incubated with HRP-conjugated secondary antibodies for 1 h at room temperature. Immunoreactive protein bands were visualized using ECL chemiluminescent substrate and imaged with a chemiluminescence imaging system. Band gray values were quantified by ImageJ software (Version 1.54f, National Institutes of Health, Bethesda, MD, USA), and the relative expression level of target proteins was normalized to the internal reference protein GAPDH.

Primary antibodies used in this experiment included: rabbit polyclonal anti-AgRP (A8044, ABClonal, Woburn, MA, USA; dilution 1:1000), anti-LC3A/LC3B (A1559, ABClonal, Woburn, MA, USA; dilution 1:1000), anti-SQSTM1/p62 (AF5312, Beyotime, China, 1:1000), anti-c-Fos (AF6489, ABClonal, dilution1:1000), anti-GABARAP (A4335, Beyotime Biotechnology, Shanghai, China; dilution 1:1000), anti-GABRG1 (PA5-119791, Thermo Fisher Scientific, Waltham, MA, USA; dilution 1:1000) and anti-GAPDH (AC027, ABclonal, Woburn, MA, USA; dilution 1:1000). The secondary antibody was HRP-labeled goat anti-rabbit IgG (H+L) (A0208, Beyotime, China).

### 2.12. Real-Time Quantitative Polymerase Chain Reaction (RT-qPCR)

Total RNA was extracted from mouse hypothalamic tissues using TRIzol reagent (Invitrogen, Carlsbad, CA, USA) according to standard operating procedures. RNA concentration and purity were detected by spectrophotometry. A total of 1 μg qualified total RNA was reverse-transcribed into cDNA using PrimeScript RT Master Mix (TaKaRa, Kusatsu, Shiga, Japan). Real-time qPCR amplification was performed with SYBR Green fluorescent quantitative PCR master mix. The specific primer sequences were as follows: AgRP (forward: 5′-CAG AAG GCA GCA GGT GAA C-3′, reverse: 5′-CAA GAG CGT GCA GAC CTG A-3′), NPY (forward: 5′-AGG CCA GAT GGA GGG TTC A-3′, reverse: 5′-GGC GTT TCT GTG CTT TCT CT-3′), and GAPDH (forward: 5′-AGG TCG GTG TGA ACG GAT TTG-3′, reverse: 5′-TGT AGA CCA TGT AGT TGA GGT CA-3′). The relative mRNA expression levels of target genes were calculated by the 2^−ΔΔCt^ method with GAPDH as the endogenous reference gene.

### 2.13. Immunofluorescence Histochemical Staining

Frozen mouse brain sections (10 μm thickness) were fixed with 4% paraformaldehyde for 15–20 min at room temperature. After PBS washing, sections were permeabilized with 0.3% Triton X-100 PBS (Sigma-Aldrich, St. Louis, MO, USA) solution for 30 min and blocked with 10% normal donkey serum for 1 h to eliminate non-specific binding. Sections were incubated with diluted primary antibodies overnight in a humidified 4 °C chamber. After full washing, fluorescent secondary antibodies (Alexa Fluor 488/555-conjugated goat anti-rabbit IgG, Thermo Fisher Scientific, Waltham, MA, USA; dilution 1:500) were added for 1 h of dark incubation at room temperature. Cell nuclei were stained with 1 μg/mL DAPI for 5 min. After final washing and anti-fade mounting, fluorescence images were captured by laser scanning confocal microscopy. Blank control groups including primary antibody omission, single secondary antibody incubation and unstained sections were set to exclude non-specific fluorescence interference.

The primary antibodies adopted in this assay were consistent with those used in western blotting, with a dilution ratio of 1:200 for most indicators and 1:500 for c-Fos antibody.

### 2.14. LC-MS Determination of GABA Concentration

The GABA content in mouse hypothalamic tissues was quantified via ultra-high-performance liquid chromatography coupled with triple quadrupole mass spectrometry (UHPLC-QqQ MS, LC-TQ5200, Guangzhou Hexin Instrument Co., Ltd., Guangzhou, China). Precisely weighed hypothalamic tissues were homogenized in 0.01% formic acid aqueous solution (containing GABA-d6 internal standard) at a mass volume ratio of 1:10. The homogenate was placed at 4 °C for 30 min and centrifuged at 12,000× *g* for 10 min at 4 °C. The supernatant was filtered through a 0.22 μm membrane and loaded into sample vials for detection.

Chromatographic separation was performed on a HILIC column with a mobile phase system of 10 mM formic acid aqueous solution (phase A) and acetonitrile (phase B) via gradient elution. Mass spectrometry detection was conducted in positive electrospray ionization mode with multiple reaction monitoring (MRM). The characteristic ion transition of GABA was set as *m*/*z* 104.1 → 87.1, and optimal collision energy was adjusted by instrument matching software. GABA standard solutions with gradient concentrations (10–50 nmol/mL) were used to establish the standard curve. Stable isotope dilution method was applied for absolute quantification: the peak area ratio of target GABA to internal standard was substituted into the standard curve to calculate tissue GABA concentration, which was finally normalized by tissue weight.

### 2.15. Autophagic Flux Detection Using Dual-Fluorescence LC3B Adenovirus

Primary neurons in logarithmic growth phase were seeded in confocal culture dishes. After cell adherence, Ad-mCherry-GFP-LC3B autophagy dual-fluorescence adenovirus (C3011-1mL, Beyotime Biotech Inc., Shanghai, China) was used for cell infection at a multiplicity of infection (MOI) of 10. Twenty-four hours after infection, the culture medium was discarded, and cells were gently rinsed with pre-warmed PBS. Laser scanning confocal microscopy was used to observe and capture fluorescence images. Yellow fluorescent puncta (co-localization of mCherry red and GFP green fluorescence) represented autophagosomes, while single red fluorescent puncta indicated autolysosomes, which were used to evaluate intracellular autophagic flux levels.

### 2.16. Cellular Immunofluorescence Staining

After completing the corresponding experimental treatments, primary neurons were rinsed with PBS and fixed with 4% paraformaldehyde on ice for 30 min. After PBS washing, cells were permeabilized and blocked with confining liquid containing 1% BSA, 0.1% Triton X-100 and 50 mM glycine for 30 min at room temperature. Cells were incubated with diluted primary antibodies (1:200 in 1% BSA solution) overnight at 4 °C. The primary antibody types were consistent with those used in tissue immunofluorescence staining. After thorough washing, cells were incubated with fluorescent secondary antibodies (1:200) for 2 h in dark conditions. Cell nuclei were stained with DAPI working solution (1:500) for 5 min. Fluorescence images were collected and analyzed by fluorescence microscopy.

### 2.17. Co-Immunoprecipitation (Co-IP)

Primary hypothalamic neurons were isolated using a commercial enzymatic digestion kit (abs955, Absin Bioscience Inc., Shanghai, China). Treated cells were rinsed with pre-cooled PBS and lysed with protease inhibitor-containing lysis buffer on ice for 30 min. Cell lysates were centrifuged at 12,000× *g* for 15 min at 4 °C, and supernatants were collected for protein concentration detection via BCA method.

An equal amount of 500 μg total protein from each group was incubated with anti-GABARAP primary antibody (18723-1-AP, Proteintech, Rosemont, IL, USA; dilution 1:1000) overnight at 4 °C with continuous rotation. Protein A agarose beads were added for further incubation for 2–4 h at 4 °C. After incubation, agarose beads were collected by low-speed centrifugation (3000× *g*, 5 min, 4 °C) and washed 3–5 times with inhibitor-containing lysis buffer. After removing the supernatant, 2× SDS-PAGE loading buffer was added, and beads were heated at 95 °C for 5 min to elute bound proteins. The eluted protein samples were detected by western blotting with anti-GABRG1 antibody to verify the interaction between GABARAP and GABA_A_R. Normal rabbit IgG was set as the negative control for immunoprecipitation.

### 2.18. Transcriptome Sequencing and Bioinformatic Analysis

To explore the molecular mechanisms underlying HFD-induced hypothalamic neuronal dysfunction and depressive-like behaviors, transcriptome sequencing was performed on hypothalamic tissue from P21 male offspring in the perinatal control diet group (C group, *n* = 3) and perinatal HFD group (H group, *n* = 3). Total RNA was extracted from isolated neurons, and mRNA was enriched using oligo(dT) magnetic beads. Transcriptome sequencing libraries were constructed through mRNA fragmentation, cDNA reverse transcription, adapter ligation and PCR amplification, and sequenced on the Illumina PE150 platform.

Raw sequencing reads were quality-controlled to remove adapter-contaminated and low-quality sequences to obtain clean reads. Qualified clean reads were aligned to the Mus musculus reference genome (GRCm38 release 95) using HISAT2 v2.1.0 software. StringTie v2.1.1 was used for transcript assembly and gene expression quantification, with gene expression levels calculated as FPKM values.

Differentially expressed genes (DEGs) between C and H groups were screened by DESeq2 v1.38.3 software with the threshold of FoldChange ≥ 1.5 and *p* < 0.05. Gene Ontology (GO) functional annotation and Kyoto Encyclopedia of Genes and Genomes (KEGG) pathway enrichment analyses were performed via the ClusterProfiler R package (version 4.14.0, Bioconductor, Seattle, WA, USA) based on hypergeometric distribution tests. Gene Set Enrichment Analysis (GSEA) was conducted using the fgsea R package with 1000 random permutations. The protein protein interaction (PPI) network of DEGs was constructed via the STRING v11.5 database and visualized by Cytoscape v3.9.1 software. Core hub genes were screened according to node degree and clustering coefficient. All transcriptomic analysis results are presented in Section 3.9.

### 2.19. Statistical Analysis

All experimental data were statistically analyzed using SPSS 26.0 software, and statistical graphs were generated by GraphPad Prism 9. The Shapiro–Wilk test was used to verify data normality, and Levene’s test was applied to detect variance homogeneity. All quantitative data are presented as mean ± standard error of the mean (SEM). For all analyses, individual offspring served as the statistical unit, and litter was treated as a blocking factor nested within maternal diet to control for within-litter correlation.

For longitudinal repeated-measures data—including food intake, energy intake, water consumption, heart rate, and systolic blood pressure measured weekly from PNW4 to PNW7—two-way repeated-measures analysis of variance (ANOVA) was performed, with maternal diet (perinatal Control vs. HFD) and adolescent diet (Control vs. HFD) as between-subject factors and time (PNW4–PNW7) as the within-subject factor. Mauchly’s test of sphericity was applied to assess the sphericity assumption; when the sphericity assumption was violated (*p* < 0.05), the Greenhouse–Geisser correction was used to adjust the degrees of freedom. Partial eta squared (*η*^2^*p*) was calculated as a measure of effect size for all ANOVA effects.

For in vivo 2 × 2 factorial experimental design data, two-way analysis of variance (ANOVA) combined with partial eta squared (*η*^2^*p*) effect size evaluation was adopted to analyze the main effects and interaction effects of perinatal and adolescent dietary interventions. For two-group comparison of in vitro cell experimental data, unpaired two-tailed Student’s *t*-test was used, and Cohen’s d was calculated to evaluate effect size. A *p*-value less than 0.05 was defined as statistically significant in all analyses.

## 3. Results

### 3.1. Effects of High-Fat Diet on Food Intake, Water Intake, Energy Intake, and Body Weight in Offspring Mice

Food intake, energy intake, and water intake were analyzed using separate two-way repeated-measures ANOVAs, each with maternal diet (perinatal Control vs. HFD) and adolescent diet (Control vs. HFD) as between-subject factors and time (PNW4–PNW7) as the within-subject factor, with sphericity assumed for all (Mauchly’s tests: *p* = 0.139, *p* = 0.164, and *p* = 0.270, respectively).

**Figure 2:**
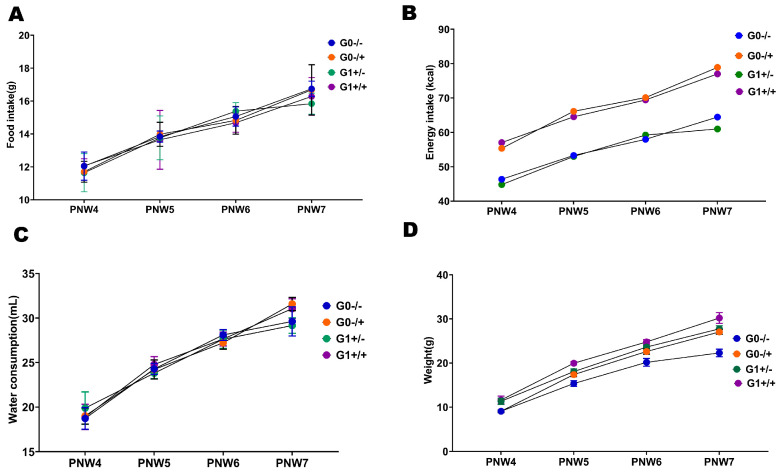
Metabolic parameters in male offspring exposed to perinatal and/or adolescent High-Fat Diet (HFD). Weekly food intake (**A**), energy intake (**B**), water consumption (**C**), and body weight (**D**) from Postnatal Week 4 to Postnatal Week 7 (PNW 4–7). Data are presented as mean ± SEM (*n* = 5 per group). G0−/−, perinatal control + adolescent control; G0−/+, perinatal control + adolescent HFD; G1+/−, perinatal HFD + adolescent control; G1+/+, perinatal HFD + adolescent HFD (as defined in Section 2.2 and Figure 1).

For food intake, a significant main effect of time was observed (*F*(3,48) = 84.387, *p* < 0.01), indicating a linear increase across the observation period (*F*(1,16) = 166.789, *p* < 0.01). No significant main effects of maternal diet (*F*(1,16) = 0.839, *p* = 0.373) or adolescent diet (*F*(1,16) = 0.070, *p* = 0.795) were detected, nor their interaction (F(1,16) = 0.096, *p* = 0.761). All time-related interactions were non-significant (time × maternal diet: *F*(3,48) = 0.609, *p* = 0.611; time × adolescent diet: *F*(3,48) = 0.467, *p* = 0.706; three-way: *F*(3,48) = 0.498, *p* = 0.686), indicating that neither perinatal nor adolescent HFD exposure affected the food intake trajectory (Figure 2A).

For energy intake, time also showed a significant main effect (*F*(3,48) = 80.148, *p* < 0.01) with a linear trend (*F*(1,16) = 162.531, *p* < 0.01). A significant main effect of adolescent diet was detected (*F*(1,16) = 159.651, *p* < 0.01, *η*^2^*p* = 0.909), with the adolescent HFD group consuming significantly more energy than the adolescent CD group (67.31 vs. 55.01 kcal/week, difference: 12.30 kcal/week). In contrast, the main effect of maternal diet (*F*(1,16) = 0.709, *p* = 0.412) and the maternal × adolescent diet interaction (*F*(1,16)= 0.042, *p* = 0.840) were not significant, nor were the time-related interactions (all *p* > 0.05) (Figure 2B).

For water intake, the main effect of time was highly significant (*F*(3,48) = 417.814, *p* < 0.01), with both linear (*F*(1,16) = 869.200, *p* < 0.01) and quadratic (*F*(1,16) = 25.968, *p* < 0.01) components. Notably, the time × adolescent diet interaction was significant (*F*(3,48) = 5.366, *p* = 0.03), with the adolescent HFD group showing a steeper increase from PNW6 to PNW7, supported by a significant linear contrast (*F*(1,16) = 5.652, *p* = 0.030). However, neither the main effect of maternal diet (*F*(1,16) = 0.002, *p* = 0.968) nor adolescent diet (*F*(1,16) = 2.729, *p* = 0.118) reached significance, and no interaction was detected (*p* > 0.05 for all other effects). Thus, adolescent HFD exposure did not affect overall water intake levels but significantly altered its temporal growth pattern, with a steeper increase during the later phase, whereas perinatal HFD exposure had no detectable effect on water intake (Figure 2C).

Body weight showed a significant time effect (*p* < 0.01), with linear (*p* < 0.01) and quadratic (*p* < 0.01) trends. Significant main effects were found for maternal diet (*F*(1,16) = 251.538, *p* < 0.01, *η*^2^*p* = 0.940) and adolescent diet (*F*(1,16) = 95.981, *p* < 0.01, *η*^2^*p* = 0.857), along with their interaction (*F*(1,16) = 4.950, *p* = 0.041). All time-related interactions were significant (all *p* < 0.01), indicating that both perinatal and adolescent HFD altered body weight growth trajectories, with combined exposure producing the highest weight and an additive/synergistic effect.

### 3.2. Effects of High-Fat Diet on Body Composition and Organ Coefficients in Offspring Mice

**Figure 3:**
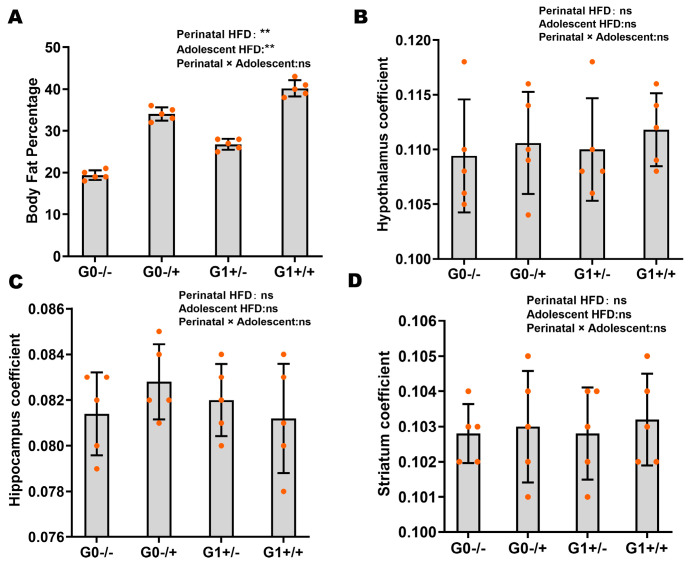
Body fat percentage and organ coefficients in Mice at Postnatal Day 45. (**A**) Body fat percentage. (**B**) Hypothalamus coefficient. (**C**) Hippocampus coefficient. (**D**) Striatum coefficient. Values are mean ± SEM, each orange dot represents an individual mouse (*n* = 5/group). Two-way ANOVA: ns, not significant; ** *p* < 0.01. G0−/−: perinatal control + adolescent control; G0−/+: perinatal control + adolescent HFD; G1+/−: perinatal HFD + adolescent control; G1+/+: perinatal HFD + adolescent HFD.

For body fat percentage (Figure 3A), both perinatal diet (*F*(1,16) = 100.522, *p* < 0.01, *η*^2^*p* = 0.863) and adolescent diet (*F*(1,16) = 426.087, *p* < 0.01, *η*^2^*p* = 0.964) exerted highly significant main effects in offspring, with adolescent diet showing a particularly large effect size. The interaction between perinatal and adolescent diets was not significant (*F*(1,16) = 0.783, *p* = 0.389, *η*^2^*p* = 0.047), indicating an additive rather than synergistic effect on body fat accumulation. The model accounted for 97.1% of the variance in body fat percentage (R^2^ = 0.971).

For organ coefficients, no significant main effects of perinatal or adolescent diets, nor their interaction, were detected for liver coefficient, hippocampal coefficient, or perirenal fat coefficient (all *p* > 0.05), suggesting that these organ-weight-to-body-weight ratios were not affected by dietary interventions.

### 3.3. Effects of High-Fat Diet on Biochemical Parameters and Blood Pressure in Offspring Mice

**Figure 4:**
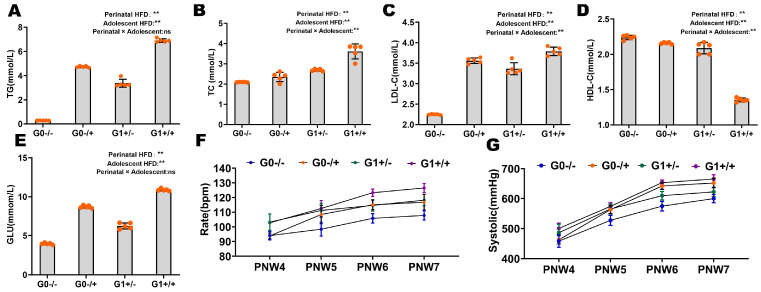
Serum biochemical parameters, heart rate, and systolic blood pressure in male offspring exposed to perinatal and/or adolescent High-Fat Diet. (**A**–**E**) Serum Triglyceride, Total Cholesterol, Low-Density Lipoprotein Cholesterol, High-Density Lipoprotein Cholesterol, and Blood Glucose at Postnatal Day 45. (**F**) Heart Rate and (**G**) Systolic blood pressure measured weekly from Postnatal Week 4 to Postnatal Week 7. Data are presented as mean ± SEM, each orange dot represents an individual mouse (*n* = 5 per group). (**A**–**E**) were analyzed using standard two-way ANOVA with maternal diet and adolescent diet as fixed factors. Heart rate and systolic blood pressure (**F**,**G**) were analyzed using two-way repeated-measures ANOVA with maternal diet and adolescent diet as between-subject factors and time Postnatal Week 4 to Postnatal Week 7 as the within-subject factor; Greenhouse–Geisser correction was applied where appropriate. Effect sizes (*η*^2^*p*) are reported for significant findings. Two-way ANOVA: ns, not significant; ** *p* < 0.01. G0−/−: perinatal control + adolescent control; G0−/+:perinatal control + adolescent HFD; G1+/−: perinatal HFD + adolescent control; G1+/+: perinatal HFD + adolescent HFD.

For serum lipid and glycemic parameters (Figure 4A–E), both perinatal and adolescent diets exerted highly significant main effects on TG, TC, LDL-C, HDL-C, and blood glucose in offspring (all *p* < 0.01, *η*^2^*p* = 0.694–0.993). Significant synergistic interactions were detected for TC (*p* < 0.01, *η*^2^*p* = 0.419) and LDL-C (*p* < 0.01, *η*^2^*p* = 0.871), indicating that combined perinatal and adolescent high-fat exposure produced an additive–synergistic effect on these two parameters, whereas TG, HDL-C, and blood glucose showed no significant interactions (all *p* > 0.05), suggesting additive rather than synergistic effects.

Heart rate was analyzed using two-way repeated-measures ANOVA (Greenhouse–Geisser correction applied, Mauchly’s *p* = 0.091). Time showed a significant main effect (*F*(2.096, 3.505) = 127.992, *p* < 0.01), with both linear (*F*(1,16) = 386.704, *p* < 0.01) and quadratic (F(1,16) = 28.459, *p* < 0.001) components. Both maternal diet (*F*(1,16) = 54.569, *p* < 0.01, *η*^2^*p* = 0.773) and adolescent diet (*F*(1,16) = 22.914, *p* < 0.01, *η*^2^*p* = 0.589) showed significant main effects, increasing heart rate by 9.38 and 6.08 bpm, respectively. A significant time × adolescent diet interaction (*F*(2.096,3.505) = 8.693, *p* < 0.001), with a significant linear contrast (*F*(1,16) = 22.944, *p* < 0.01), indicated that adolescent HFD accelerated the heart rate increase from PNW5 onward, whereas the time × maternal diet interaction was not significant (*p* = 0.835). No significant maternal × adolescent diet interaction (*p* = 0.477) or three-way interaction (*p* = 0.113) was detected (Figure 4F).

Systolic blood pressure was similarly analyzed (Box’s test *p* = 0.040, Greenhouse–Geisser applied, Mauchly’s *p* = 0.084). Time showed a significant main effect (*F*(2.174,4.778) = 1166.264, *p* < 0.01), with linear, quadratic, and cubic trends (all *p* < 0.05). Significant main effects were observed for both maternal diet (*F*(1,16) = 15.642, *p* = 0.01, *η*^2^*p* = 0.494) and adolescent diet (*F*(1,16) = 29.268, *p* < 0.01, *η*^2^*p* = 0.647), increasing blood pressure by 24.60 and 33.65 mmHg, respectively. A significant time × adolescent diet interaction (*F*(2.174,4.778) = 26.072, *p* < 0.01) with a significant linear contrast (F(1,16) = 55.276, *p* < 0.01) indicated that adolescent HFD accelerated blood pressure rise from PNW5. The time × maternal diet interaction approached significance (*p* = 0.094), and the three-way interaction was significant (*F*(2.174,4.778) = 4.043, *p* = 0.012), indicating that combined exposure produced a unique nonlinear pattern—a steep surge followed by plateau—beyond additive effects. No significant maternal × adolescent diet interaction was detected for overall levels (*p* = 0.330) (Figure 4G).

### 3.4. Effects of High-Fat Diet on Behavioral Performance in Offspring Mice

**Figure 5:**
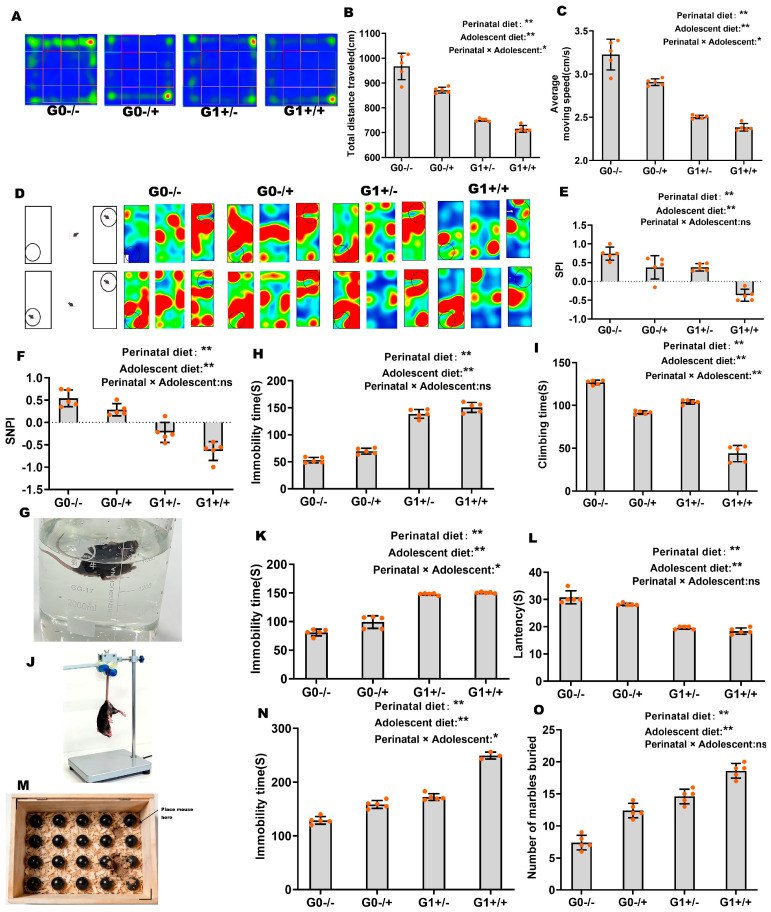
Behavioral performance at Postnatal Day 45. (**A**) Representative heatmaps of locomotor activity in the open field test. (**B**,**C**) Total distance traveled and average speed. (**D**) Representative heatmaps in the three-chamber social interaction test. (**E**,**F**) Social preference index and social novelty preference index. (**G**) Schematic of the forced swim test. (**H**,**I**) Immobility and climbing times. (**J**) Schematic of the tail-suspension test. (**K**,**L**) Latency to immobility and immobility time. (**M**) Schematic of the marble-burying test. (**N**,**O**) Immobility time and number of marbles buried. Values are mean ± SEM, each orange dot represents an individual mouse (*n* = 5/group). Two-way ANOVA: ns, not significant; * *p* < 0.05; ** *p* < 0.01. G0−/−: perinatal control + adolescent control; G0−/+: perinatal control + adolescent HFD; G1+/−: perinatal HFD + adolescent control; G1+/+: perinatal HFD + adolescent HFD.

For the open field test (Figure 5A–C), both perinatal and adolescent diets significantly decreased total distance traveled in the central area (perinatal: *F*(1,16) = 214.942, *p* < 0.01, *η*^2^*p* = 0.931; adolescent: *F*(1,16) = 27.179, *p* < 0.01, *η*^2^*p* = 0.629) and average movement speed (perinatal: *F*(1,16) = 217.229, *p* < 0.01, *η*^2^*p* = 0.931; adolescent: *F*(1,16) = 27.176, *p* < 0.01, *η*^2^*p* = 0.629), with significant synergistic interactions detected for both parameters (central distance: *F*(1,16) = 5.496, *p* < 0.05, *η*^2^*p* = 0.256; speed: *F*(1,16) = 5.615, *p* < 0.05, *η*^2^*p* = 0.260), indicating that combined exposure produced an additive–synergistic suppression of exploratory activity.

For the three-chamber social interaction test (Figure 5D–F), both perinatal and adolescent diets significantly reduced the social preference index (SPI; perinatal: *F*(1,16) = 37.672, *p* < 0.01, *η*^2^*p* = 0.702; adolescent: *F*(1,16) = 37.830, *p* < 0.01, *η*^2^*p* = 0.703) and the social novelty preference index (SNPI; perinatal: *F*(1,16) = 95.965, *p* < 0.01, *η*^2^*p* = 0.857; adolescent: *F*(1,16) = 15.136, *p* < 0.01, *η*^2^*p* = 0.486). The interaction for SPI approached significance (*F*(1,16) = 4.424, *p* = 0.052, *η*^2^*p* = 0.217), suggesting a potential synergistic trend, whereas the SNPI interaction was not significant (*F*(1,16) = 0.863, *p* = 0.367, *η*^2^*p* = 0.051), indicating additive effects.

In the forced swim test (Figure 5G,H), both perinatal and adolescent diets significantly increased immobility time (perinatal: *F*(1,16) = 669.448, *p* < 0.01, *η*^2^*p* = 0.977; adolescent: *F*(1,16) = 19.407, *p* < 0.01, *η*^2^*p* = 0.548) with no significant interaction (*F*(1,16) = 0.554, *p* = 0.467, *η*^2^*p* = 0.033), and significantly decreased climbing time (perinatal: *F*(1,16) = 232.479, *p* < 0.01, *η*^2^*p* = 0.936; adolescent: *F*(1,16) = 424.494, *p* < 0.01, *η*^2^*p* = 0.964) with a significant synergistic interaction (*F*(1,16) = 29.151, *p* < 0.01, *η*^2^*p* = 0.646).

In the tail-suspension test (Figure 5K,L), both perinatal and adolescent diets significantly decreased latency to immobility (perinatal: *F*(1,16) = 294.000, *p* < 0.01, *η*^2^*p* = 0.948; adolescent: *F*(1,16) = 9.627, *p* < 0.01, *η*^2^*p* = 0.376) with no significant interaction (*F*(1,16) = 1.307, *p* = 0.270, *η*^2^*p* = 0.076), and significantly increased total immobility time (perinatal: *F*(1,16) = 438.903, *p* < 0.01, *η*^2^*p* = 0.965; adolescent: *F*(1,16) = 13.545, *p* < 0.01, *η*^2^*p* = 0.458) with a significant synergistic interaction (*F*(1,16) = 7.619, *p* = 0.014, *η*^2^*p* = 0.323).

In the marble-burying test (Figure 5N,O), both perinatal and adolescent diets significantly increased immobility time (perinatal: *F*(1,16) = 107.332, *p* < 0.01, *η*^2^*p* = 0.870; adolescent: *F*(1,16) = 63.382, *p* < 0.01, *η*^2^*p* = 0.798) with a significant synergistic interaction (*F*(1,16) = 7.930, *p* < 0.05, *η*^2^*p* = 0.331), and significantly increased the number of marbles buried (perinatal: *F*(1,16) = 129.310, *p* < 0.01, *η*^2^*p* = 0.890; adolescent: *F*(1,16) = 42.506, *p* < 0.01, *η*^2^*p* = 0.727) with no significant interaction (*F*(1,16) = 0.023, *p* = 0.881, *η*^2^*p* = 0.001), indicating additive rather than synergistic effects on marble burying.

### 3.5. Effects of High-Fat Diet on Hypothalamic AgRP-Associated Protein and mRNA Levels

**Figure 6:**
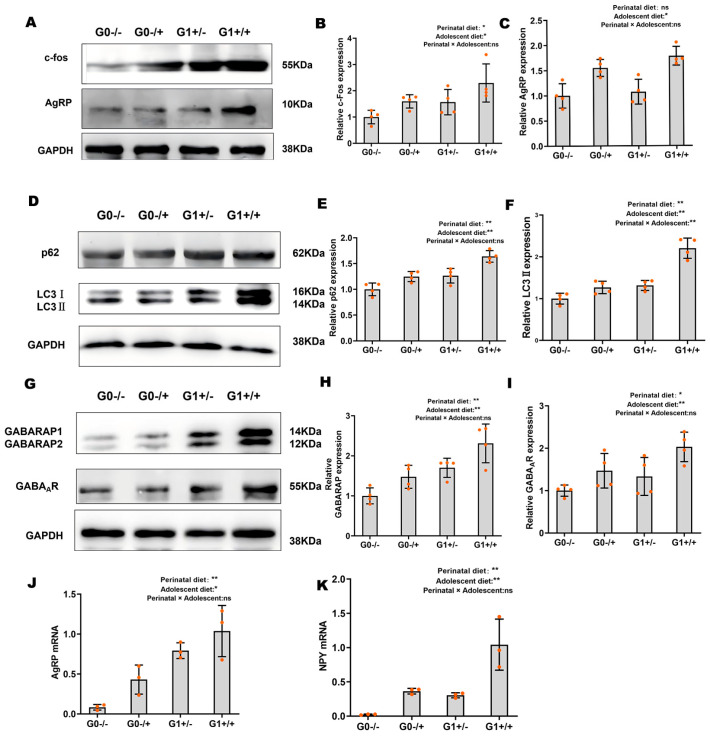
Expression of AgRP-related proteins and mRNA in the hypothalamus of male offspring mice at postnatal day 45. (**A**) Representative western blot bands of AgRP and c-Fos. (**B**) Quantification of c-Fos protein expression. (**C**) Quantification of AgRP protein expression. (**D**) Representative western blot bands of p62 and LC3-II. (**E**) Quantification of p62 protein expression. (**F**) Quantification of LC3-II protein expression. (**G**) Representative western blot bands of GABARAP and GABA_A_R. (**H**) Quantification of GABARAP expression. (**I**) Quantification of GABA_A_R protein expression. (**J**) Relative AgRP mRNA expression in the paraventricular nucleus. (**K**) Relative NPY mRNA expression in the paraventricular nucleus. AgRP: Agouti-Related Peptide;c-Fos: Proto-oncogene c-Fos;p62: Sequestosome-1;LC3-II: Microtubule-associated protein 1 light chain 3 alpha;GABARAP: Gamma-aminobutyric acid receptor-associated protein;GABA_A_R: Gamma-aminobutyric acid type A receptor;NPY: Neuropeptide YValues are expressed as mean ± SEM, each orange dot represents an individual mouse (*n* = 3–4 per group). Data were analyzed by 2 × 2 factorial ANOVA with effect size estimation. Two-way ANOVA results for main effects and interactions are indicated in each panel: ns, non-significant; * *p* < 0.05; ** *p* < 0.01. G0−/−: perinatal control + adolescent control; G0−/+: perinatal control + adolescent HFD; G1+/−: perinatal HFD + adolescent control; G1+/+: perinatal HFD + adolescent HFD.

Western blot analysis (Figure 6A–I) revealed that both perinatal and adolescent diets significantly upregulated c-Fos protein expression (perinatal: *F*(1,12) = 7.205, *p* < 0.05, *η*^2^*p* = 0.375; adolescent: *F*(1,12) = 7.869, *p* < 0.05, *η*^2^*p* = 0.396) with no significant interaction (*p* = 0.787). For AgRP, a highly significant main effect of adolescent diet was observed (*F*(1,12) = 34.856, *p* < 0.01, *η*^2^*p* = 0.744), whereas perinatal diet and the interaction were not significant. Both perinatal and adolescent diets significantly upregulated p62 (perinatal: *F*(1,12) = 29.996, *p* < 0.01, *η*^2^*p* = 0.714; adolescent: *F*(1,12) = 27.084, *p* < 0.01, *η*^2^*p* = 0.693) and GABARAP (perinatal: *F*(1,12) = 22.793, *p* < 0.01, *η*^2^*p* = 0.655; adolescent: *F*(1,12) = 11.376, *p* <0.01, *η*^2^*p* = 0.487), with no significant interactions for either marker (*p* > 0.05). For LC3-II, both perinatal and adolescent diets exerted highly significant main effects (perinatal: *F*(1,12) = 56.599, *p* < 0.01, *η*^2^*p* = 0.825; adolescent: *F*(1,12) = 48.218, *p* < 0.01, *η*^2^*p* = 0.801), along with a significant synergistic interaction (*F*(1,12) = 13.929, *p* < 0.01, *η*^2^*p* = 0.537). Similarly, both perinatal and adolescent diets significantly upregulated GABA_A_R (perinatal: *F*(1,12) = 6.385, *p* < 0.05, *η*^2^*p* = 0.347; adolescent: *F*(1,12) = 10.777, *p* < 0.01, *η*^2^*p* = 0.473), with no significant interaction (*p* = 0.532).

RT-qPCR analysis (Figure 6J,K) showed that both perinatal and adolescent diets significantly upregulated AgRP mRNA (perinatal: *F*(1,8) = 35.404, *p* < 0.01, *η*^2^*p* = 0.816; adolescent: *F*(1,8) = 7.153, *p* < 0.05, *η*^2^*p* = 0.472) and NPY mRNA (perinatal: *F*(1,8) = 19.459, *p* < 0.01, *η*^2^*p* = 0.709; adolescent: *F*(1,8) = 24.601, *p* < 0.01, *η*^2^*p* = 0.755) in the paraventricular nucleus, with no significant interactions for either gene (*p* > 0.05).

### 3.6. Effects of Early-Life High-Fat Diet on Hypothalamic Autophagy–GABARAP Signaling

**Figure 7:**
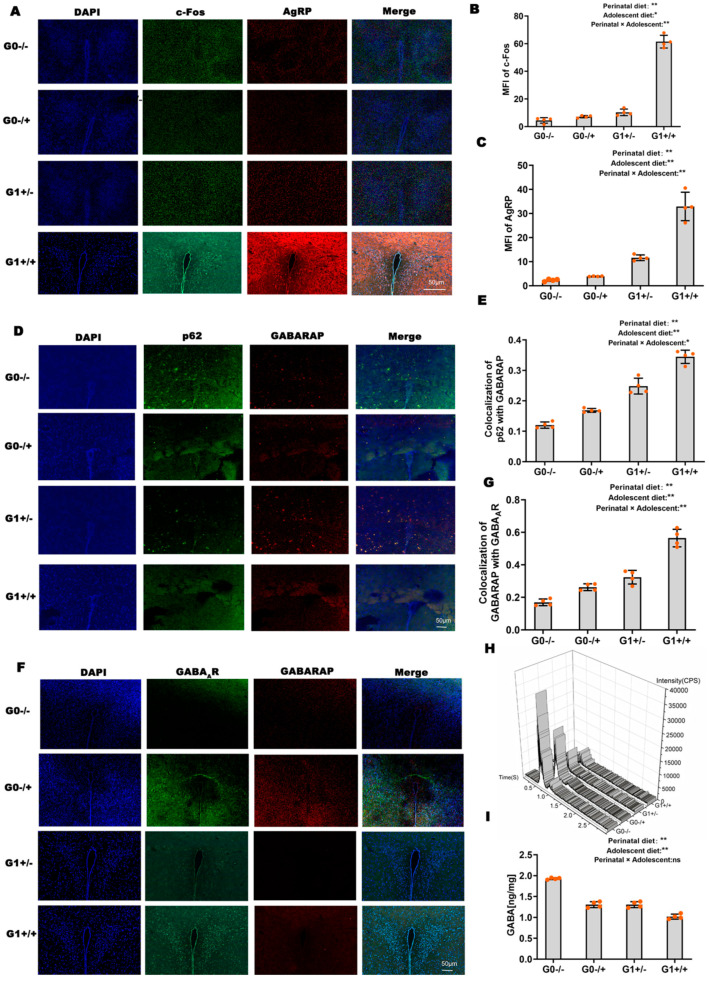
Immunofluorescence analysis of AgRP neuron-related proteins and GABA concentration in the hypothalamus of male offspring mice at postnatal day 45. (**A**) Representative immunofluorescence images of c-Fos (red) and AgRP (green) in the paraventricular nucleus. (**B**) Quantification of c-Fos fluorescence intensity. (**C**) Quantification of AgRP fluorescence intensity. (**D**) Representative immunofluorescence images of p62 (red) and GABARAP (green) in the PVN. (**E**) Co-localization coefficient of p62 and GABARAP. (**F**) Representative immunofluorescence images of GABARAP (red) and GABA_A_R (green) in the PVN. (**G**) Co-localization coefficient of GABARAP and GABA_A_R. (**H**) Representative extracted ion chromatogram of GABA. (**I**) Quantitative analysis of GABA concentration in hypothalamic tissue. Scale bar = 50 μm (*n* = 4 per group). AgRP: Agouti-Related Peptide; c-Fos: Proto-oncogene c-Fos; p62: Sequestosome-1; GABARAP: Gamma-aminobutyric acid receptor-associated protein; GABA_A_R: Gamma-aminobutyric acid type A receptor; GABA:Gamma-aminobutyric acid. Values are expressed as mean ± SEM, each orange dot represents an individual mouse. Data were analyzed by 2 × 2 factorial ANOVA with effect size estimation. Two-way ANOVA results for main effects and interactions are indicated in each panel: ns, non-significant; * *p* < 0.05; ** *p* < 0.01. G0−/−: perinatal control + adolescent control; G0−/+: perinatal control + adolescent HFD; G1+/−: perinatal HFD + adolescent control; G1+/+: perinatal HFD + adolescent HFD.

Immunofluorescence staining of the paraventricular nucleus (PVN; Figure 7B,C) revealed that both perinatal and adolescent diets significantly increased c-Fos fluorescence intensity (perinatal: *F*(1,12) = 160.533, *p* < 0.01, *η*^2^*p* = 0.930; adolescent: *F*(1,12) = 57.385, *p* < 0.01, *η*^2^*p* = 0.827), with a significant synergistic interaction (*F*(1,12) = 42.454, *p* < 0.01, *η*^2^*p* = 0.780). For AgRP fluorescence intensity, both perinatal (*F*(1,12) = 35.404, *p* < 0.01, *η*^2^*p* = 0.816) and adolescent (*F*(1,12) = 7.153, *p* = 0.028, *η*^2^*p* = 0.472) diets significantly increased its expression, with no significant interaction (*p* = 0.658).

For co-localization analysis (Figure 7E–G), both perinatal and adolescent diets significantly increased the p62/GABARAP co-localization coefficient (perinatal: *F*(1,12) = 284.660, *p* < 0.01, *η*^2^*p* = 0.960; adolescent: *F*(1,12) = 64.362, *p* < 0.01, *η*^2^*p* = 0.843), with no significant interaction (*p* = 0.627). The GABARAP/GABA_A_R co-localization coefficient was also significantly increased by both perinatal (*F*(1,12) = 151.632, *p* < 0.01, *η*^2^*p* = 0.927) and adolescent (*F*(1,12) = 80.887, *p* < 0.01, *η*^2^*p* = 0.871) diets, with a significant synergistic interaction (*F*(1,12) = 15.774, *p* < 0.01, *η*^2^*p* = 0.568).

Hypothalamic GABA concentration measured by mass spectrometry (Figure 7I) was significantly decreased by both perinatal (*F*(1,12) = 623.152, *p* < 001, *η*^2^*p* = 0.981) and adolescent (*F*(1,12) = 102.684, *p* < 0.01, *η*^2^*p* = 0.895) diets, with no significant interaction (*p* = 0.390). The model accounted for 98.4% of the variance (R^2^ = 0.984).

### 3.7. Effects of High-Fat Diet on Hypothalamic Autophagic Flux in Offspring Mice

**Figure 8:**
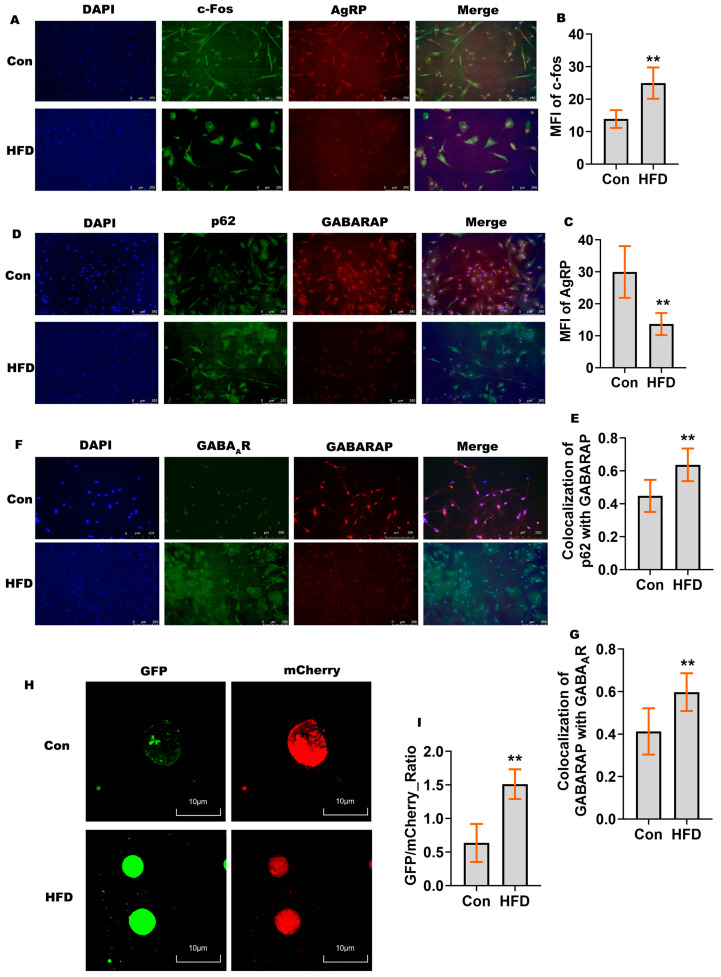
Autophagic flux and protein expression in primary hypothalamic neurons. (**A**,**B**) Autophagic flux assessed by Ad-mCherry-GFP-LC3B reporter (scale bar = 250 μm); GFP/mCherry ratio. (**C**–**E**) Immunofluorescence of c-Fos (red) and AgRP (green); fluorescence intensity quantifications (scale bar = 10 μm). (**F**,**G**) Immunofluorescence of p62 (red) and GABARAP (green); co-localization coefficient. (**H**,**I**) Immunofluorescence of GABARAP (red) and GABAAR (green); co-localization coefficient. Ad-mCherry-GFP-LC3B: Adenovirus encoding tandem fluorescent-tagged mCherry-GFP-LC3B; GFP: Green Fluorescent Protein; mCherry: mCherry Fluorescent Protein; LC3B: Microtubule-associated protein 1 light chain 3 beta; c-Fos: Proto-oncogene c-Fos; AgRP: Agouti-Related Peptide; p62: Sequestosome-1; GABARAP: Gamma-aminobutyric acid receptor-associated protein; GABA_A_R: Gamma-aminobutyric acid type A receptor. Values are mean ± SEM (*n* = 4 independent cultures/group). ** *p* < 0.01 versus Con group (Student’s *t*-test). Con: control diet offspring neurons + DMSO; HFD: HFD offspring neurons + DMSO.

Primary hypothalamic neurons were isolated from male offspring of dams fed either a control diet (Con) or a perinatal high-fat diet (HFD). Autophagic flux was assessed using the GFP-mCherry-LC3 reporter, and protein expression was examined by immunofluorescence (Figure 8).

The HFD group displayed a significantly higher GFP/mCherry ratio compared with the Con group (unpaired *t*-test, t(10) = 10.88, *p* < 0.01, Cohen’s d = 2.93; Figure 8B), indicating that perinatal HFD exposure suppressed autophagic flux and promoted autophagosome accumulation in these cultures. c-Fos fluorescence intensity was significantly increased in the HFD group (t(10) = 12.61, *p* < 0.01, Cohen’s d = 2.82; Figure 8D), whereas AgRP fluorescence intensity was significantly decreased (t(10) = −11.72, *p* < 0.01, Cohen’s d = 2.46; Figure 8E). The co-localization coefficient of p62 and GABARAP was significantly higher in the HFD group than in the Con group (t(10) = 10.88, *p* < 0.01, Cohen’s d = 2.46; Figure 8G), suggesting increased intracellular proximity or association between the autophagic substrate p62 and GABARAP. Similarly, the co-localization coefficient of GABARAP and GABA_A_R was significantly increased in the HFD group (t(10) = 9.22, *p* < 0.01, Cohen’s d = 2.09; Figure 8I), indicating an increased co-localization of GABARAP with GABA_A_R under conditions of impaired autophagic flux. All Cohen’s d values exceeded 0.8, confirming that the observed effects were of large to very large magnitude.

### 3.8. Effects of Rapamycin on Autophagy–GABARAP Pathway Proteins in Primary Hypothalamic Neurons

**Figure 9:**
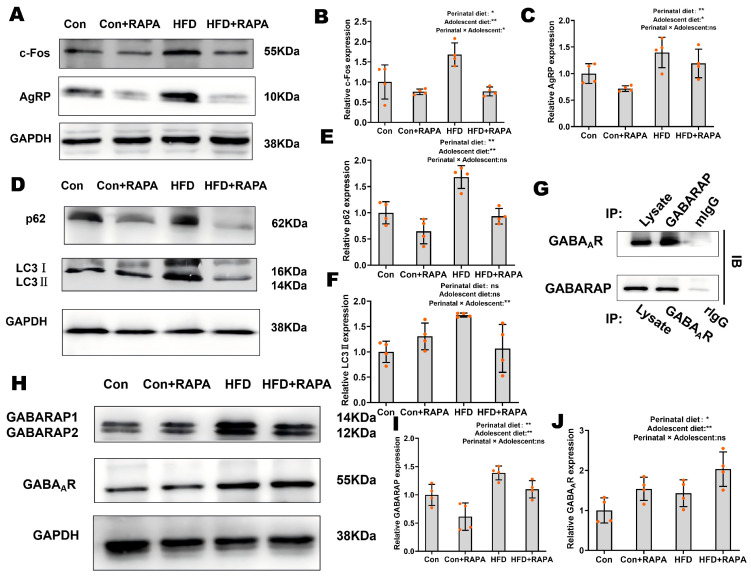
Effects of rapamycin on protein expression and GABARAP–GABA_A_R interaction in primary hypothalamic neurons. (**A**–**C**) Representative blots and quantifications of AgRP and c-Fos. (**D**–**F**) Representative blots and quantifications of p62 and LC3-II. (**G**–**I**) Representative blots and quantifications of GABARAP and GABA_A_R. (**J**) Co-IP showing GABARAP–GABA_A_R interaction. AgRP: Agouti-Related Peptide; c-Fos: Proto-oncogene c-Fos; p62: Sequestosome-1; LC3-II: Microtubule-associated protein 1 light chain 3 alpha; GABARAP: Gamma-aminobutyric acid receptor-associated protein; GABA_A_R: Gamma-aminobutyric acid type A receptor. Values are mean ± SEM, each orange dot represents an individual mouse (*n* = 4 independent cultures/group). Two-way ANOVA: ns, not significant; * *p* < 0.05; ** *p* < 0.01. Con: control diet offspring neurons + DMSO; Con + RAPA: control diet offspring neurons + rapamycin; HFD: HFD offspring neurons + DMSO, and HFD + RAPA: HFD offspring neurons + rapamycin.

Primary hypothalamic neurons were divided into four groups: Con (neurons from control diet offspring, standard culture), Con+RAPA (Con treated with rapamycin), HFD (neurons from perinatal HFD offspring, standard culture), and HFD+RAPA (HFD treated with rapamycin). Protein expression was assessed by Western blot, and protein–protein interaction was verified by co-immunoprecipitation (Co-IP) (Figure 9).

For c-Fos (Figure 9B), a significant main effect of perinatal diet (*F*(1,12) = 6.826, *p* < 0.05, *η*^2^*p* = 0.363) and rapamycin treatment (*F*(1,12) = 19.282, *p* < 0.01, *η*^2^*p* = 0.616) was observed, with a significant interaction between the two factors (*F*(1,12) = 6.418, *p* < 0.05, *η*^2^*p* = 0.348), indicating that rapamycin reversed the HFD-induced upregulation of c-Fos.

For AgRP (Figure 9C), perinatal HFD significantly upregulated its expression (*F*(1,12) = 15.848, *p* < 0.01, *η*^2^*p* = 0.569), while rapamycin significantly downregulated it (*F*(1,12) = 4.989, *p* < 0.05, *η*^2^*p* = 0.294), with no significant interaction (*p* = 0.731).

For p62 (Figure 9E), both perinatal HFD (*F*(1,12) = 22.349, *p* < 0.01, *η*^2^*p* = 0.651) and rapamycin (*F*(1,12) = 28.850, *p* < 0.01, *η*^2^*p* = 0.706) exerted significant main effects, with the interaction approaching significance (*F*(1,12) = 3.647, *p* = 0.080, *η*^2^*p* = 0.233), suggesting a potential synergistic trend.

For LC3-II (Figure 9F), neither main effect reached significance (*p* > 0.05), but a significant interaction was detected (*F*(1,12) = 11.004, *p* < 0.01, *η*^2^*p* = 0.478), indicating that the effect of perinatal HFD on LC3-II expression was dependent on rapamycin treatment.

For GABARAP (Figure 9H), both perinatal HFD (*F*(1,12) = 23.037, *p* < 0.01, *η*^2^*p* = 0.658) and rapamycin (*F*(1,12) = 13.812, *p* < 0.01, *η*^2^*p* = 0.535) significantly regulated its expression, with no significant interaction (*p* = 0.603). Similarly, for GABA_A_R (Figure 9I), both perinatal HFD (*F*(1,12) = 7.150, *p* < 0.05, *η*^2^*p* = 0.373) and rapamycin (*F*(1,12) = 10.857, *p* < 0.01, *η*^2^*p* = 0.475) significantly regulated its expression, with no significant interaction (*p* = 0.859).

Co-IP confirmed a direct protein interaction between GABARAP and GABA_A_R (Figure 9J). This binding interaction, together with the observed changes in protein expression and co-localization, suggests a potential role for GABARAP in modulating GABA_A_R protein homeostasis in the context of autophagy modulation.

### 3.9. Transcriptomic Profiling of Hypothalamic Tissue from Offspring Exposed to a Perinatal High-Fat Diet

**Figure 10:**
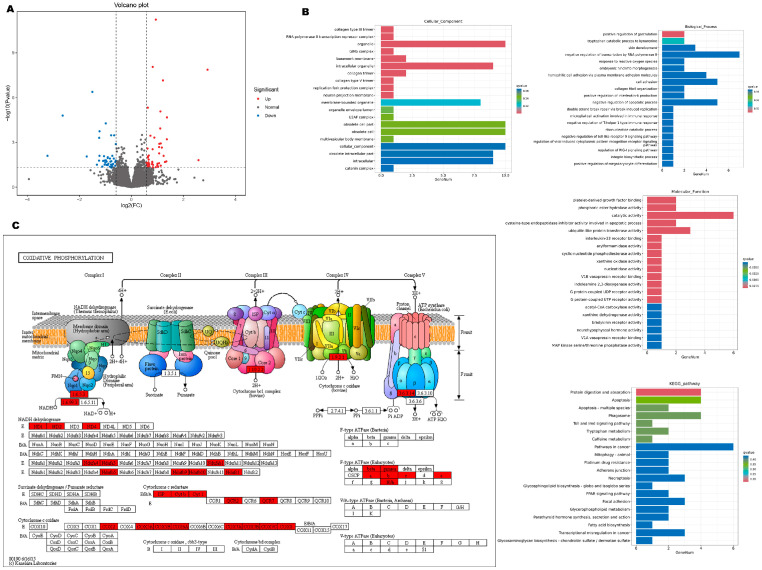
Transcriptomic profiling and functional enrichment analysis of differentially expressed genes in hypothalamic tissue from newborn offspring mice. Total RNA was extracted from the hypothalami of male newborn offspring born to dams fed a perinatal control diet or a perinatal high-fat diet and subjected to transcriptomic sequencing. (**A**) Volcano plot of differentially expressed genes (DEGs). Each dot represents a gene. The *x*-axis represents log_2_-transformed fold change (log_2_FC), and the *y*-axis represents –log_10_-transformed *p* value. Red dots indicate significantly upregulated genes (log_2_FC > 1 and *p* < 0.05), blue dots indicate significantly downregulated genes (log_2_FC < −1 and *p* < 0.05), and gray dots indicate non-significant genes. (**B**) Functional enrichment analysis of DEGs, showing significantly enriched Gene Ontology (GO) terms and KEGG pathways. (**C**) KEGG pathway annotation illustrating the expression of core subunits in the oxidative phosphorylation (OXPHOS) pathway. Relative to the control group, enzymes marked with red boxes are associated with upregulated genes, green boxes with downregulated genes, and blue boxes with both up- and downregulated genes; the numbers within the boxes represent enzyme commission (EC) numbers. The entire pathway consists of complex biochemical reactions catalyzed by multiple enzymes, and enzymes associated with DEGs are highlighted in different colors.

To investigate the molecular mechanisms underlying perinatal high-fat diet (HFD)-induced changes, transcriptome sequencing was performed on hypothalamic tissue isolated from male offspring of the perinatal HFD group (H) and the perinatal control diet group (C). Using a significance threshold of *p* < 0.05, 87 differentially expressed genes (DEGs) were identified, including 46 upregulated and 41 downregulated genes (Figure 10A). Among the DEGs related to autophagy/lipophagy, oxidative stress, neuronal activity, and synaptic function, the lipophagy marker *Plin4* (log_2_FC = 2.93), oxidative stress regulator *Txnip* (log_2_FC = 0.63), and metabolic stress-related *Angptl4* (log_2_FC = 1.12) were significantly upregulated in the H group, whereas the neuronal activity regulator *Npas4* (log_2_FC = −1.32) and depression-related *Avp* (log_2_FC = −3.22) were significantly downregulated. No transcriptional changes were observed in GABA receptor subunits or core canonical autophagy genes.

Gene Ontology (GO) and Kyoto Encyclopedia of Genes and Genomes (KEGG) enrichment analyses (Figure 10B) showed that the DEGs were significantly enriched in biological processes and cellular components such as ubiquitin-like protein transferase activity, neuronal projection membrane, multivesicular body membrane, response to reactive oxygen species, and tryptophan–kynurenine metabolism. Within the enriched KEGG pathways, mitophagy and tryptophan metabolism were significantly overrepresented, with the oxidative phosphorylation (OXPHOS) pathway displaying the highest enrichment. Core subunits of Complexes I–IV (e.g., ND1, ND2, COX4, and ATP synthase subunits) were all significantly upregulated in the H group, suggesting that perinatal HFD induces mitochondrial energy metabolic remodeling in the hypothalamus.

**Figure 11:**
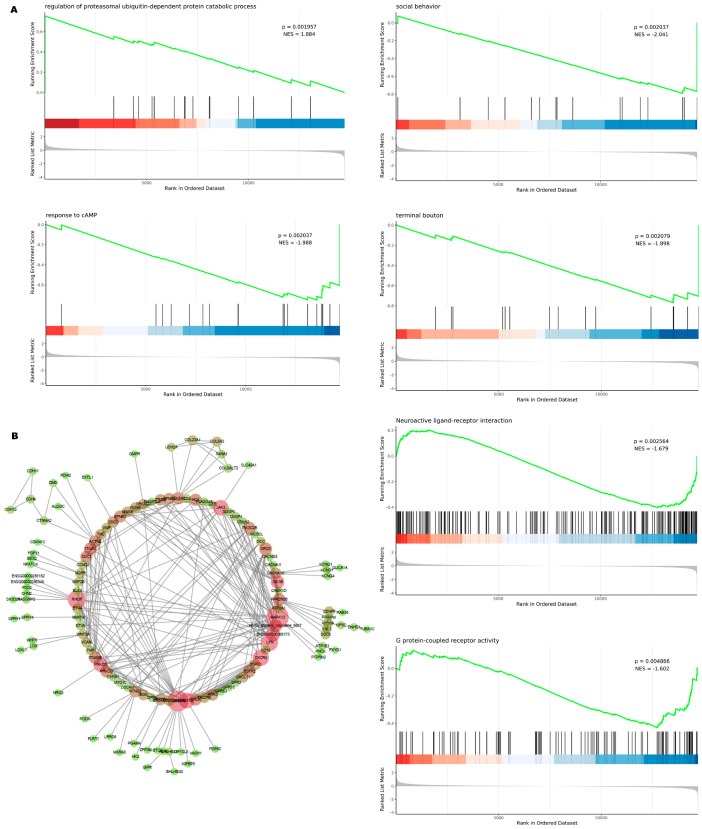
Protein–protein interaction network and gene set enrichment analysis in hypothalamic tissue from newborn offspring mice. (**A**) Gene Set Enrichment Analysis (GSEA) curves for representative gene sets. The green curve represents the running enrichment score at each position; its peak denotes the ES value for the corresponding GO term/KEGG pathway. A positive ES indicates that genes enriched before the peak are core genes of the gene set and the function is upregulated, whereas a negative ES indicates that genes enriched after the peak are core genes and the function is downregulated. The height of black vertical bars represents the log2FC values of genes within the gene set, corresponding to the red/blue annotation in the middle. Normalized enrichment score (NES) and nominal *p* value are annotated in the plots. (**B**) Protein–protein interaction (PPI) network constructed based on differentially expressed genes (DEGs). Nodes represent proteins, and edges represent interactions. Node size is proportional to degree (i.e., more connected edges result in a larger node). Node color scales from green (low) to red (high), representing the clustering coefficient; a redder color indicates better connectivity among neighboring nodes of a given node.

Gene Set Enrichment Analysis (GSEA; Figure 11A) revealed significant upregulation of the ubiquitin–proteasome-dependent protein catabolic process (normalized enrichment score [NES] = 1.884), while pathways related to social behavior, cAMP response, synaptic bouton, neuroactive ligand–receptor interaction, and GPCR activity were significantly downregulated (NES = −2.041, −1.988, −1.898, −1.679, and −1.602, respectively). These data indicate that perinatal HFD activates the ubiquitin–proteasome system while being associated with suppressed neuronal function and cAMP/GPCR signaling at the transcriptional level.

A protein–protein interaction (PPI) network constructed from the DEGs (Figure 11B) identified hub proteins with high degree and clustering coefficient, including RHOF, MAPK13, JAK3, DRD2, and CXCR4, which serve as central nodes linking cytoskeletal remodeling, signal transduction, neuroinflammation, and dopamine signaling.

Taken together, these transcriptomic data reveal that perinatal HFD exposure is associated with activation of mitophagy and the ubiquitin–proteasome system, inhibition of cAMP/GPCR signaling and synaptic function-related pathways, and altered expression of hub proteins such as RHOF and MAPK13 in hypothalamic tissue. However, the absence of transcriptional changes in GABA receptor subunits and core autophagy genes suggests that the observed alterations in GABA_A_R and GABARAP levels are likely regulated at the post-transcriptional or post-translational level. The functional implications of these transcriptional changes for GABA_A_R protein trafficking or membrane localization in specific neuronal subtypes were not directly assessed in this study and require further investigation using cell-type-specific approaches.

## 4. Discussion

The present study demonstrates that perinatal and adolescent HFD exposure induces metabolic disturbances, depressive-like behaviors, and multifaceted alterations in hypothalamic autophagic flux and GABARAP–GABA_A_R protein interactions in offspring mice. Specifically, HFD exposure led to: (1) accumulation of autophagic markers p62 and LC3-II with a significant synergistic interaction for LC3-II (*F*(1,12) = 13.929, *p* < 0.01); (2) increased GABARAP levels and GABARAP–GABA_A_R co-localization in the hypothalamic paraventricular nucleus, accompanied by reduced GABA_A_R protein abundance and decreased hypothalamic GABA concentration; (iii) elevated c-Fos expression, indicating heightened hypothalamic neuronal activity; and (iv) transcriptional reprogramming involving 87 DEGs enriched in mitophagy, oxidative phosphorylation, and ubiquitin–proteasome pathways. These findings were further corroborated in primary hypothalamic neuronal cultures, where rapamycin partially reversed HFD-induced protein expression changes, and Co-IP confirmed a direct GABARAP–GABA_A_R interaction.

### 4.1. Autophagic Flux Impairment and GABARAP–GABAAR Association

The observed accumulation of p62 (perinatal: *F*(1,12) = 29.996, *p* < 0.01; adolescent: *F*(1,12) = 27.084, *p* < 0.01) and LC3-II (perinatal: *F*(1,12) = 56.599, *p* < 0.01; adolescent: *F*(1,12) = 48.218, *p* < 0.01), together with the significantly increased GFP/mCherry ratio in primary cultures (t(10) = 10.88, *p* < 0.01, Cohen’s d = 2.93), indicates that early-life HFD exposure suppresses autophagic flux in the hypothalamus. This is consistent with previous reports linking nutritional excess to autophagy dysfunction in metabolic tissues [34,35]. Notably, the significant synergistic interaction for LC3-II (*F*(1,12) = 13.929, *p* < 0.01) suggests that concurrent perinatal and adolescent HFD exposure produces an additive effect on autophagosome accumulation beyond that of either period alone.

GABARAP, as a member of the Atg8 family, participates in autophagosome formation and maturation [25,37]. Both perinatal (*F*(1,12) = 22.793, *p* < 0.01) and adolescent *(F*(1,12) = 11.376, *p* < 0.01) diets significantly upregulated GABARAP levels, with no significant interaction (*p* > 0.05). The p62/GABARAP co-localization coefficient was markedly increased by both perinatal (*F*(1,12) = 284.660, *p* < 0.01, *η*^2^*p* = 0.960) and adolescent (*F*(1,12) = 64.362, *p* < 0.01, *η*^2^*p* = 0.843) diets, suggesting that impaired autophagic flux may lead to aberrant accumulation of GABARAP-containing autophagic structures.

Importantly, the GABARAP/GABA_A_R co-localization coefficient was significantly increased by both perinatal (*F*(1,12) = 151.632, *p* < 0.01, *η*^2^*p* = 0.927) and adolescent (*F*(1,12) = 80.887, *p* < 0.01, *η*^2^*p* = 0.871) diets, with a significant synergistic interaction (*F*(1,12) = 15.774, *p* < 0.01, *η*^2^*p* = 0.568). This was accompanied by reduced GABA_A_R protein abundance (perinatal: *F*(1,12) = 6.385, *p* < 0.05, *η*^2^*p* = 0.347; adolescent: *F*(1,12) = 10.777, *p* < 0.01, *η*^2^*p* = 0.473) and decreased hypothalamic GABA concentration (perinatal: *F*(1,12) = 623.152, *p* < 0.01, *η*^2^*p* = 0.981; adolescent: *F*(1,12) = 102.684, *p* < 0.01, *η*^2^*p* = 0.895; R^2^ = 0.984). Co-IP confirmed a direct protein–protein interaction between GABARAP and GABA_A_R in primary hypothalamic cultures (Figure 9J). However, it is critical to emphasize that co-localization and Co-IP data alone do not establish the functional consequences of this association. Increased co-localization could reflect accumulation of GABARAP–GABA_A_R complexes in autophagic compartments, altered trafficking dynamics, or simply increased protein abundance of both partners without functional significance. Direct assessment of GABA_A_R surface expression (e.g., via surface biotinylation), membrane localization (e.g., via subcellular fractionation or immunoelectron microscopy), or receptor function (e.g., via patch-clamp electrophysiology) would be required to determine whether the observed GABARAP–GABA_A_R association affects GABA_A_R availability at the plasma membrane or synaptic sites. Such experiments were beyond the scope of the present study and represent important directions for future investigation.

### 4.2. Neuronal Hyperactivity and GABAergic Dysfunction

Both perinatal (*F*(1,12) = 7.205, *p* < 0.05) and adolescent (*F*(1,12) = 7.869, *p* < 0.05) HFD exposure significantly upregulated c-Fos protein expression, with immunofluorescence further confirming increased c-Fos intensity (perinatal: *F*(1,12) = 160.533, *p* < 0.01; adolescent: *F*(1,12) = 57.385, *p* < 0.01) and a significant synergistic interaction (*F*(1,12) = 42.454, *p* < 0.01, *η*^2^*p* = 0.780). These findings indicate that early-life HFD exposure promotes hypothalamic neuronal hyperactivity. Given that GABA is the primary inhibitory neurotransmitter in the hypothalamus, the observed reduction in GABA concentration—accounting for 98.4% of the variance (R^2^ = 0.984)—together with reduced GABA_A_R protein abundance, likely contributes to disinhibition of hypothalamic neurons. This is consistent with the classical notion that GABAergic signaling dysfunction is a hallmark of depression [18,19,38,39,40]. However, the relative contributions of altered GABA synthesis, release, or receptor availability to the observed neuronal hyperactivity cannot be distinguished from the present data.

For AgRP, a highly significant main effect of adolescent diet was observed for protein expression (*F*(1,12) = 34.856, *p* < 0.01, *η*^2^*p* = 0.744), while both perinatal (*F*(1,8) = 35.404, *p* < 0.01) and adolescent (*F*(1,8) = 7.153, *p* < 0.05) diets upregulated AgRP mRNA in the paraventricular nucleus. The discordance between AgRP protein and mRNA regulation across perinatal versus adolescent exposure periods suggests complex, time-dependent post-transcriptional regulation that warrants further investigation.

### 4.3. Transcriptomic Reprogramming and Its Relationship to Protein-Level Changes

Transcriptome sequencing of hypothalamic tissue identified 87 DEGs (46 upregulated, 41 downregulated) in perinatally HFD-exposed offspring. The lipophagy marker Plin4 (log_2_FC = 2.93), oxidative stress regulator Txnip (log_2_FC = 0.63), and metabolic stress-related Angptl4 (log_2_FC = 1.12) were significantly upregulated, whereas the neuronal activity regulator Npas4 (log_2_FC = −1.32) and depression-related Avp (log_2_FC = −3.22) were significantly downregulated. Enrichment analyses revealed significant overrepresentation of mitophagy, oxidative phosphorylation (OXPHOS), and ubiquitin–proteasome pathways. Core subunits of Complexes I–IV (e.g., ND1, ND2, COX4, ATP synthase subunits) were all significantly upregulated, suggesting mitochondrial energy metabolic remodeling in the hypothalamus following perinatal HFD exposure. GSEA further revealed upregulation of the ubiquitin–proteasome-dependent protein catabolic process (NES = 1.884), alongside downregulation of pathways related to social behavior (NES = −2.041), cAMP response (NES = −1.988), synaptic bouton (NES = −1.898), neuroactive ligand–receptor interaction (NES = −1.679), and GPCR activity (NES = −1.602). These data indicate that perinatal HFD activates protein degradation systems while broadly suppressing neuronal signaling pathways at the transcriptional level.

Notably, no transcriptional changes were observed in GABA receptor subunits or core canonical autophagy genes. This dissociation between transcriptional and protein-level changes indicates that the observed alterations in GABA_A_R and GABARAP abundance and co-localization are likely regulated at the post-transcriptional or post-translational level. This is consistent with the known role of GABARAP as a protein–protein interaction scaffold that modulates GABA_A_R stability and trafficking through binding rather than transcriptional regulation [23,24,41,42,43,44]. The PPI network identified hub proteins including RHOF, MAPK13, JAK3, DRD2, and CXCR4, which may serve as central nodes linking cytoskeletal remodeling, signal transduction, neuroinflammation, and dopamine signaling in response to early-life nutritional stress.

### 4.4. Rapamycin Reverses HFD-Induced Molecular Alterations in Primary Hypothalamic Neurons

In primary hypothalamic neuronal cultures, rapamycin treatment significantly reversed HFD-induced upregulation of c-Fos (interaction: *F*(1,12) = 6.418, *p* < 0.05, *η*^2^*p* = 0.348) and modulated the expression of autophagy-related proteins. For p62, both perinatal HFD (*F*(1,12) = 22.349, *p* < 0.01, η^2^p = 0.651) and rapamycin (*F*(1,12) = 28.850, *p* < 0.01, *η*^2^*p* = 0.706) exerted significant main effects, with the interaction approaching significance (*F*(1,12) = 3.647, *p* = 0.080, *η*^2^*p* = 0.233). For LC3-II, a significant interaction was detected (*F*(1,12) = 11.004, *p* < 0.01, *η*^2^*p* = 0.478), indicating that the effect of perinatal HFD on LC3-II expression was dependent on rapamycin treatment. Both GABARAP (HFD: *F*(1,12) = 23.037, *p* < 0.01; rapamycin: *F*(1,12) = 13.812, *p* < 0.01) and GABA_A_R (HFD: *F*(1,12) = 7.150, *p* < 0.05; rapamycin: *F*(1,12) = 10.857, *p* < 0.01) were significantly regulated by both factors, with no significant interactions. Rapamycin, a well-established mTOR inhibitor, promotes autophagy by suppressing mTOR signaling, thereby enhancing autophagic flux. In this context, rapamycin treatment effectively attenuated HFD-induced alterations in autophagy-related proteins, suggesting that mTOR-dependent autophagic dysfunction contributes to the molecular changes associated with perinatal HFD exposure [44,45,46,47,48,49].

These in vitro findings support the hypothesis that pharmacological activation of autophagy via mTOR inhibition can partially reverse HFD-induced molecular alterations. However, it should be noted that these experiments were conducted in mixed primary hypothalamic cultures, and the effects of rapamycin were not examined in vivo. Whether pharmacological or genetic modulation of autophagy can rescue the depressive-like phenotypes in HFD-exposed offspring remains to be tested.

### 4.5. Limitations and Future Directions

Several important limitations should be acknowledged. First, the experimental materials used in this study were whole hypothalamic tissue and mixed primary hypothalamic neuronal cultures, not purified AgRP neurons. Cell-type-specific approaches—such as AgRP neuron-specific genetic manipulation, translating ribosome affinity purification (TRAP), or single-cell RNA sequencing—would be required to determine whether the observed effects are cell-autonomous or reflect broader hypothalamic responses.

Second, as noted above, increased co-localization and Co-IP of GABARAP and GABA_A_R do not provide direct evidence for altered receptor trafficking, membrane localization, or synaptic anchoring. Functional assays, including surface biotinylation to quantify plasma membrane GABA_A_R levels, immunohistochemistry with extracellular epitope tags to visualize surface receptors, and electrophysiological recordings to assess GABA_A_R-mediated currents, are necessary to determine whether the observed molecular changes translate into altered receptor function.

Third, while rapamycin treatment partially reversed HFD-induced protein changes in primary cultures, the effects of rapamycin were not examined in vivo. Whether pharmacological or genetic modulation of autophagy can rescue the depressive-like phenotypes in HFD-exposed offspring remains to be tested.

Fourth, the present study focused on male offspring. Given established sex differences in both metabolic and psychiatric disorders [50,51,52,53], future studies should include female offspring to determine whether similar mechanisms operate in both sexes.

Fifth, the transcriptomic analysis was performed on whole hypothalamic tissue from newborn offspring, whereas the protein-level analyses were conducted at P45. The temporal gap between these measurements limits our ability to draw direct conclusions about the causal relationships between transcriptional reprogramming and later protein-level changes. Longitudinal studies tracking both transcriptomic and proteomic changes across developmental time points would be valuable.

## 5. Conclusions

In conclusion, this study demonstrates that perinatal and adolescent HFD exposure in mice is associated with metabolic disturbances, depressive-like behaviors, impaired hypothalamic autophagic flux, increased GABARAP–GABA_A_R co-localization and binding, reduced GABA_A_R protein abundance, and elevated hypothalamic neuronal activity. Transcriptomic analysis revealed activation of mitophagy and ubiquitin proteasome pathways, alongside downregulation of neuronal signaling pathways, without transcriptional changes in GABA receptor subunits or core autophagy genes. The direct protein–protein interaction between GABARAP and GABA_A_R, confirmed by Co-IP, together with the observed co-localization changes, suggests a potential link between autophagy GABARAP dysregulation and GABA_A_R protein homeostasis in the hypothalamus. However, the functional consequences of these molecular changes—particularly regarding GABA_A_R surface expression, membrane localization, synaptic anchoring, or receptor function—remain to be directly demonstrated. Future studies employing cell-type-specific genetic manipulations, surface receptor labeling, and electrophysiological recordings are warranted to establish causal relationships and to evaluate the autophagy–GABARAP axis as a potential therapeutic target for depression associated with early-life metabolic stress.

## Data Availability

All data in the figures are included in the manuscript. Data can be obtained from the authors upon request.

## Supplementary Materials

The following supporting information can be downloaded at: https://www.mdpi.com/article/10.3390/nu18142312/s1, Table S1 The changes in body composition of each group of mice.
