# Supplementary material for: Early-Life High-Fat Diet Impairs Autophagy and Reduces GABAA Receptor Expression in Hypothalamic Neurons, Promoting Depressive-like Behaviors in Offspring Mice
Source: Nutrients. 2026 Jul 14;18(14):2312. doi: 10.3390/nu18142312 (PMC13431247; doi:10.3390/nu18142312)
Supplement: Supplementary file 1 [file nutrients-18-02312-s001.zip › nutrients-4393636-supplementary.pdf]

Table S1. The changes in body composition of each group of mice

Group comparison	Weight (g)	Fat Mass (g)	Lean Mass (g)	Free Water Mass (g)	Total Water Mass (g)
G0-/-	22.45	4.2655	18.1845	4.7427	12.4352
G0-/-	23.76	4.752	19.008	5.078	14.1265
G0-/-	22.78	4.7838	17.9962	6.0121	15.0123
G0-/-	25.17	4.5306	20.6394	4.899	11.4783
G0-/-	26.13	4.9647	21.1653	5.6575	13.7598
G0-/+	30.21	9.6672	20.5428	5.2032	14.894
G0-/+	31.78	10.4874	21.2926	6.784	14.989
G0-/+	33.47	11.7145	21.7555	6.9801	15.0340
G0-/+	34.65	11.781	22.869	5.8395	15.1028
G0-/+	36	12.96	23.04	5.7493	15.2946
G1+/-	30.87	7.7175	23.1525	6.0182	16
G1+/-	30.58	7.9508	22.6292	6.0328	16.0476
G1+/-	31.48	8.4996	22.9804	5.8731	14.9057
G1+/-	32.07	8.9796	23.0904	5.743	15.037
G1+/-	29.2	8.468	20.732	5.8934	14.879
G1+/+	33.62	13.1784	20.4416	5.7384	16.0347
G1+/+	35.72	14.817	20.903	5.805	16.348
G1+/+	33.42	14.2308	19.1892	5.905	15.4753
G1+/+	35.45	14.09	21.36	6.07	15.85
G1+/+	34.2	13.0528	21.1472	6.1087	15.9756
